# Assembly formation of minor dihydrosphingomyelin in sphingomyelin-rich ordered membrane domains

**DOI:** 10.1038/s41598-020-68688-7

**Published:** 2020-07-16

**Authors:** Masanao Kinoshita, Takumi Kyo, Nobuaki Matsumori

**Affiliations:** Department of Chemistry, Graduate School of Science, Kyushu University, Motooka 744, Nishi-ku, Fukuoka, 819-0395 Japan

**Keywords:** Membrane biophysics, Membrane structure and assembly, Sphingolipids

## Abstract

The lipidome of mammalian cells not only contain sphingomyelin (SM) but also, as a minor component, dihydrosphongomyelin (DHSM), in which the double bond at C4–C5 in the sphingosine base is reduced to a single-bond linkage. It has been indicated that DHSM forms ordered domains more effectively than SM due to its greater potential to induce intermolecular hydrogen bonds. However, direct information on partition and dynamic behaviors of DHSM in raft-like liquid-ordered (L_o_) and non-raft-like liquid-disordered (L_d_) phase-segregated membranes has been lacking. In the present study, we prepared fluorescent derivatives of DHSM and compared their behaviors to those of fluorescent SM and phosphatidylcholine (PC) derivatives. Fluorescence microscopy showed that DHSM is more preferentially localized to the L_o_ domains in the L_o_/L_d_ phase-segregated giant unilamellar vesicles than SM and PC. Most importantly, diffusion coefficient measurements indicated that DHSM molecules form DHSM-condensed assembly inside the SM-rich L_o_ domain of the SM/dioleoylphosphatidylcholine/cholesterol system even when DHSM accounts for 1–3.3 mol% of total lipids. Such heterogeneous distribution of DHSM in the SM-rich L_o_ domains was further confirmed by inter-lipid FRET experiments. This study provides new insights into the biological functions and significance of minor component DHSM in lipid rafts.

## Introduction

**Figure 1:**
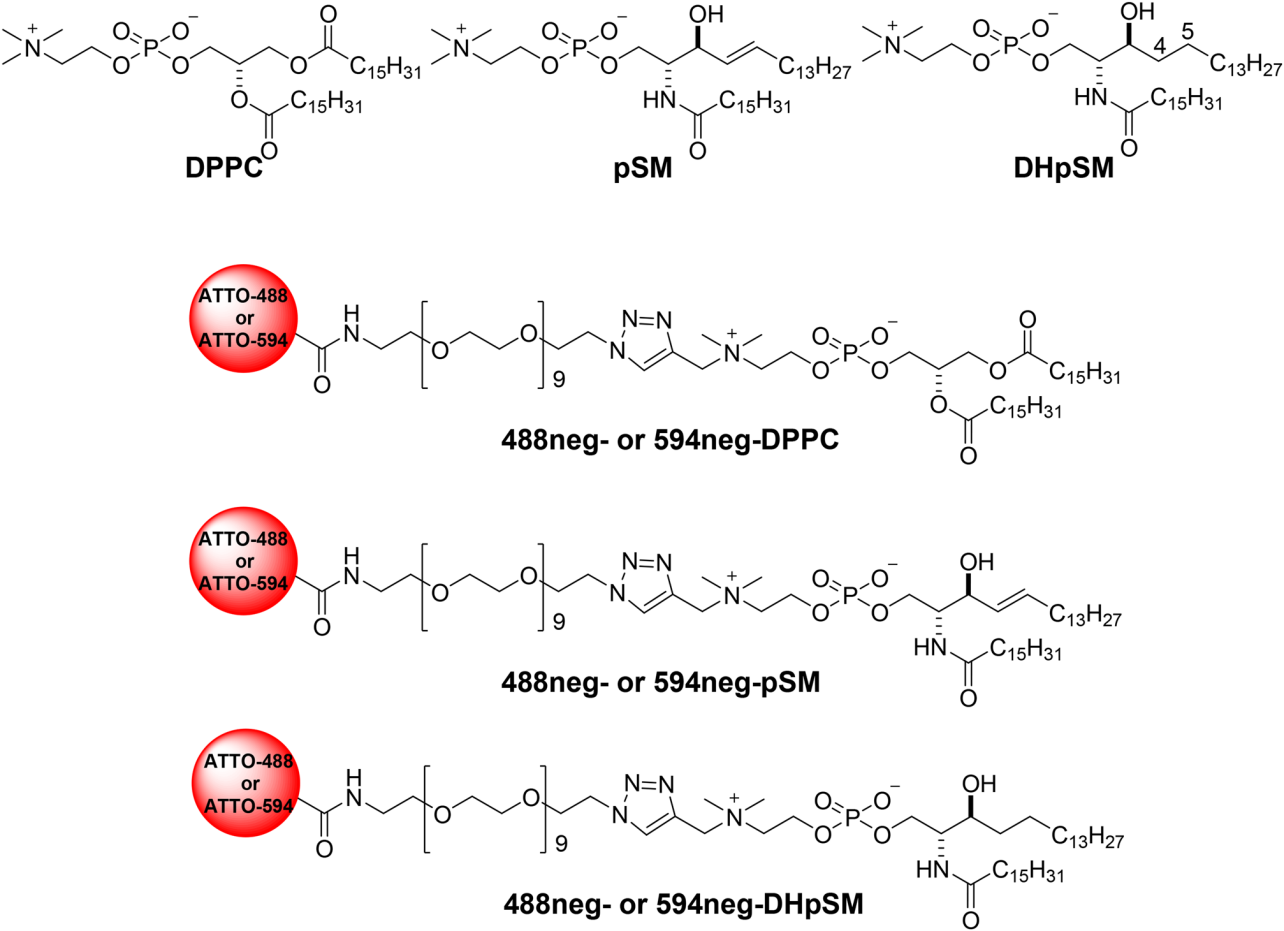
Structures of dipalmitoylphosphatidylcholine (DPPC), palmitoylsphingomyelin (pSM), palmitoyldihydrosphingomielin (DHpSM), and their fluorescent derivatives, 488neg- and 594neg-DPPCs (neg-DPPCs), 488neg- and 594neg-pSMs (neg-pSMs), and 488neg- and 594neg-DHpSMs (neg-DHpSMs). While fluorescently labeled stearoyl-SMs (C18:0) were previously reported^10^, the lengths of the lipid acyl chains in this study were unified as palmitoylate (C16:0).

In cell membranes, lipid rafts construct a platform for various important biological processes such as signal transduction and viral infections^1,2^. This finding has boosted interest in the heterogeneous distribution of sphingolipids (SLs), represented by sphingomyelin (SM, Fig. 1), in cell membranes, because distribution of SLs likely provides pivotal clues to understanding the mechanism underlying raft formation and raft-based biological events. The physicochemical properties of lipid rafts have been examined with artificial membrane systems consisting of SM, cholesterol (chol), and unsaturated phospholipids such as dioleoylphosphatidylcholine (DOPC) or palmitoyloleoylphosphatidylcholine (POPC), because these mixtures undergo macroscopic phase separation between raft-like liquid-ordered (L_o_) and non-raft-like liquid-disordered (L_d_) domains^3–8^. In order to understand dynamic and partition behaviors of SMs in artificial and biological membranes, fluorescent probes of SM have been essential and indispensable. However, direct information regarding diffusion and localization of SM has been difficult to obtain, owing to the lack of appropriate SM probes; the currently available fluorescent SM analogs, in which fluorophores are attached to the acyl chain, favor L_d_ domains rather than L_o_ domains in phase-separated artificial membranes^9^. Recently, we developed excellent fluorescent derivatives of SM: 488neg-SM and 594neg-SM (inclusively termed neg-SMs; Fig. 1)^10^, which reproduce partition and dynamic behaviors of native SM and thus enable the visualization of SM in artificial and biological membranes^10–12^.

Dihydrosphingomyelin (DHSM, Fig. 1), in which the double bond between C4 and C5 of SM is reduced to a single bond, is a minor constituent of sphingolipids. In fact, it accounts for only 5–10% of all SMs in normal cells^13,14^. On the other hand, DHSM is the major phospholipid of the human lens membranes^15^, and a substantial amount of DHSM is contained in the HIV viral membrane^16^. Notably, several recent studies have indicated that DHSM has a higher potential to provide intermolecular hydrogen bonds than the usual type of SM^17,18^. For example, palmitoyl-SM (pSM) shows a main transition at 41.2 °C in its pure bilayer form^19^, while its dihydro-congener (DHpSM) shows this at 47.7 °C^17,20,21^. Concerning the domain formation of DHSM, fluorescent quenching measurements showed that DHpSM/chol bilayers form more ordered domains than pSM/chol bilayers^22^. In addition, DHSMs are more likely than SMs to undergo lateral phase separation from DOPC membranes^23^ and may thus, contribute to the formation of laterally condensed domains in biomembranes^24^. These results considered in combination suggest that DHSM forms more condensed and ordered domains than SM via effective hydrogen bond formation.

However, the underlying reason for cell membranes containing DHSM is currently not fully understood. Hence, to scrutinize the membrane properties of DHSM and its functions in lipid rafts, the development of new fluorescent derivatives that reproduce the behaviors of DHSM is desirable. In this study, applying the procedure used for the development of fluorescent SMs^10^, we prepared novel fluorescent derivatives of DHSM (488neg-DHpSM and 594neg-DHpSM, inclusively termed neg-DHpSMs; Fig. 1) and compared their properties with those of fluorescent SM and PC to discuss the functional role of DHSM in ordered membranes.

## Results and discussion

### Preparation of fluorescent lipid analogs

**Figure 2:**
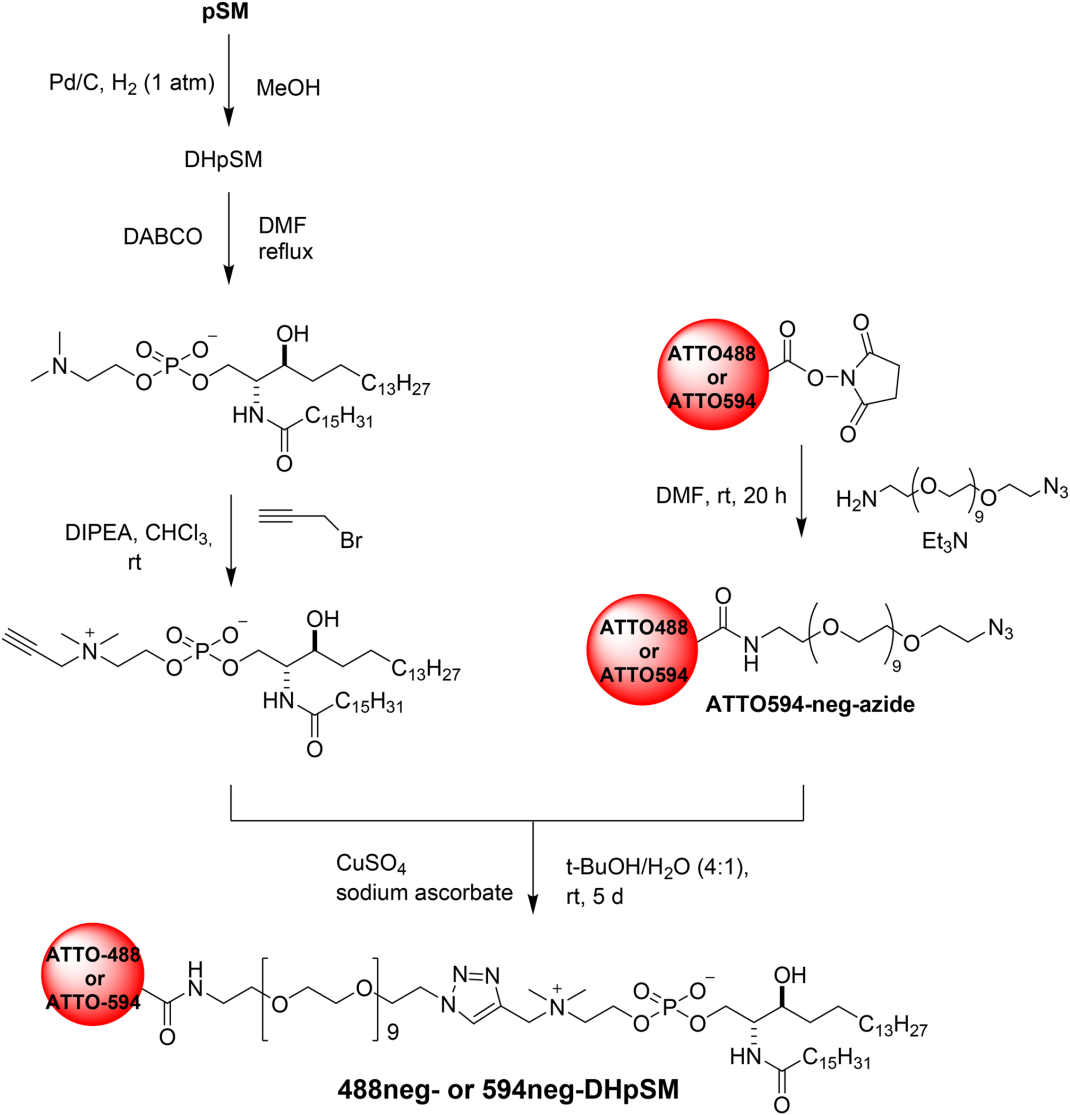
Preparation of 488neg- and 594neg-DHpSM.

As mentioned above, we reported fluorescent SMs (Fig. 1) that show quite similar partition and dynamic behaviors of native SM^10^. Our strategy for their development was to connect hydrophilic fluorophores ATTO488 and ATTO594 to the polar head of SM via a hydrophilic nonaethylene glycol (neg) linker so as to place the fluorophores away from the membrane surface toward the bulk aqueous phase. Hence, based on this strategy, we first prepared DHpSM via catalytic hydrogenation of pSM and synthesized two kinds of fluorescent DHpSM, 488neg- and 594neg-DHpSM (Fig. 2). In this study, we adopted palmitoyl (C16:0) congeners of SM and DHSM (pSM and DHpSM, respectively) for preparation of fluorescent derivatives while stearoylated SM was used in our previous report^10^. For comparison, we also prepared 488neg- and 594neg-DPPCs in a similar manner.

### Partition and dynamic behavior of fluorescent lipids in ternary GUVs

**Figure 3:**
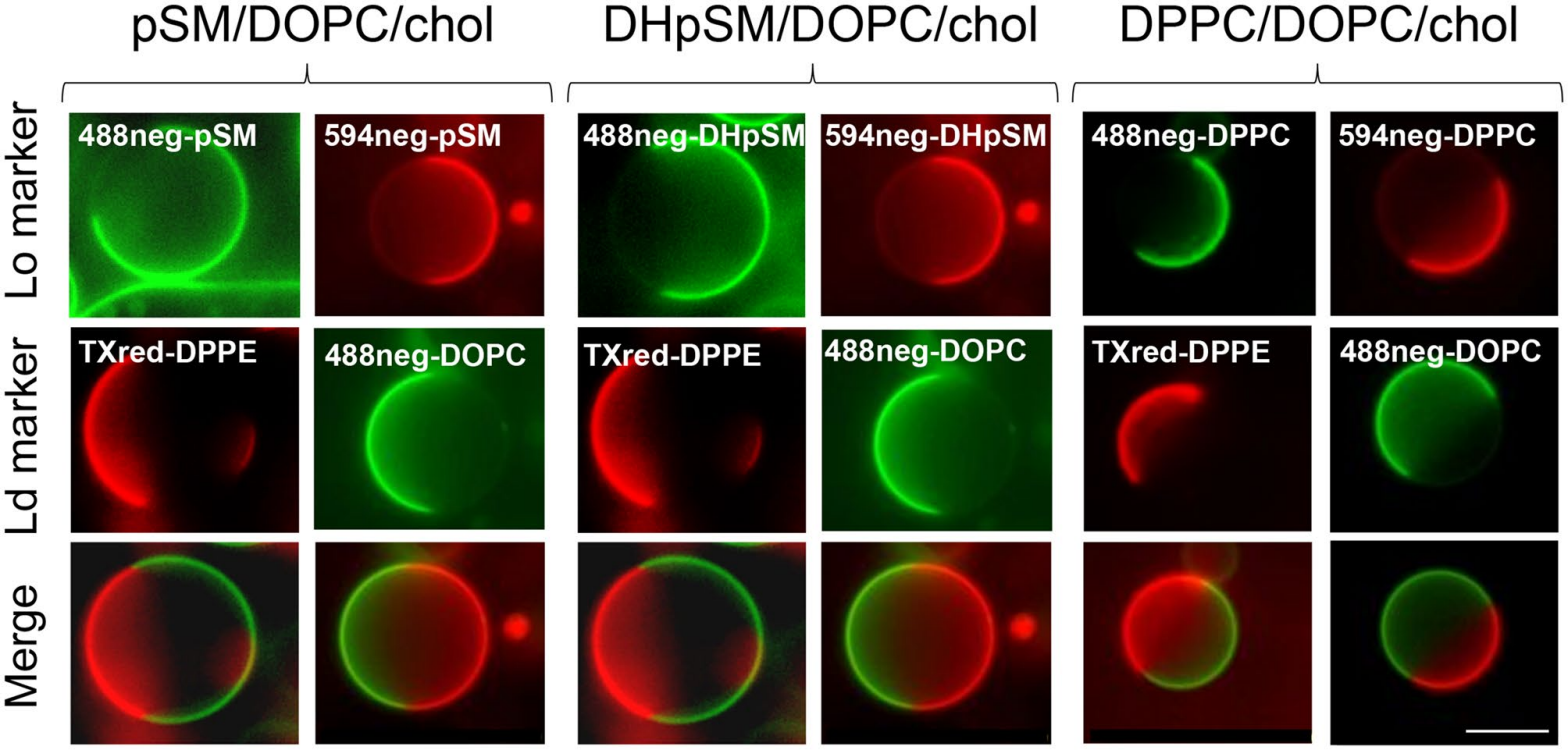
Fluorescence micrographs of ternary-component GUVs consisting of (left) pSM/DOPC/chol, (center) DHpSM/DOPC/chol, and (right) DPPC/DOPC/chol at a molar ratio of 1:1:1. These samples contain 0.2 mol% neg-pSMs, neg-DHpSM, or neg-DPPC. The L_d_ phase was labeled by 0.2 mol% 488neg-DOPC or TXred-DPPE. All of these samples underwent phase separation between the L_o_ and L_d_ domains. A bar indicates 10 μm. The brightness and contrast have been enhanced for clarity.

First, we examined the distribution of neg-DHpSMs, neg-pSMs, and neg-DPPCs in the L_o_/L_d_ phase-separated giant unilamellar vesicles (GUVs) using fluorescence microscopy (Fig. 3). Here, we prepared three kinds of ternary GUVs consisting of DHpSM/DOPC/chol, pSM/DOPC/chol, and DPPC/DOPC/chol (1:1:1 molar ratio), which contain neg-DHSMs, neg-pSMs, and neg-DPPCs, respectively. The L_d_ phases were labeled by TexasRed-DPPE (TXred-DPPE) or 488neg-DOPC^10,25,26^.

As a result, all neg-pSMs, neg-DHpSMs, and neg-DPPCs showed contrasting partition behavior compared to the L_d_ markers (top and middle panels in Fig. 3), demonstrating that these fluorescent lipid analogs were favorably incorporated into the L_o_ domains. We also randomly selected 20–30 GUVs for each sample and calculated the average intensity ratios of the L_o_ to the L_d_ domains (Table 1). Notably, DHpSM is most preferentially localized in the L_o_ domains, followed by pSM. The lowest preference of DPPC for the L_o_ domains seems reasonable, since SMs bearing additional amide and hydroxy groups have higher ability to form intermolecular hydrogen bonds than PC^27^. To further explore the difference between DHpSM and pSM, we compared diffusion coefficients of 488neg-pSM and 488neg-DHpSM in the GUVs by fluorescence correlation spectroscopy (FCS) (Table 1). As expected, 488neg-DHpSM in DHpSM/DOPC/chol ternary GUVs showed a significantly slower diffusion in the L_o_ domain compared with 488neg-SM in pSM/DOPC/chol GUVs. These distribution and diffusion data can be accounted for by the notion that DHSM forms more ordered and condensed domains than SM due to its stronger intermolecular interaction, thus preferentially accumulating in the L_o_ domain.

**Table 1:** Properties of fluorescent lipids in consistent and inconsistent ternary GUVs.

Fluorescent lipids	Composition of GUV molar ratio = 1:1:1	Distribution ratio of L_o_/L_d_^a^	Diffusion coefficient (µm^2^/s)^b^	
			L_o_ domains	L_d_ domains
488neg-DHpSM	DHpSM/DOPC/chol	4.4 ± 0.4	0.30 ± 0.03	5.0 ± 0.4
488neg-pSM	pSM/DOPC/chol	3.0 ± 0.1	0.72 ± 0.06	5.3 ± 0.5
488neg-DPPC	DPPC/DOPC/chol	2.0 ± 0.2	0.80 ± 0.04	5.3 ± 0.5
488neg-DHpSM	pSM/DOPC/chol	4.2 ± 0.3	0.61 ± 0.07	5.6 ± 0.4
488neg-pSM	DHpSM/DOPC/chol	3.6 ± 0.2	0.36 ± 0.07	5.0 ± 0.3

Distribution ratio and diffusion coefficients are presented as mean ± SE. Representative FCS curves are shown in Figs. S1 and S2.

^a^The content of fluorescent lipid was 0.2 mol% of total lipids.

^b^The content of fluorescent lipid was 0.002 mol% of total lipids.

Next, we further explored the distribution of 488neg-pSM and 488neg-DHpSM in inconsistent GUV systems: namely, 488neg-pSM (0.2 mol% of total lipids) in DHpSM/DOPC/chol GUVs, and 488neg-DHpSM (0.2 mol% of total lipids) in pSM/DOPC/chol GUVs (Table 1). Interestingly, the inter-phase distribution ratio of 488neg-DHpSM in pSM/DOPC/chol (4.2 ± 0.3) is significantly higher than that of 488neg-pSM in pSM/DOPC/chol (3.0 ± 0.1), and is similar to that of 488neg-DHpSM in DHpSM/DOPC/chol (4.4 ± 0.4; Table 1). These results clearly show that neg-DHpSM is effectively concentrated in the L_o_ domains, regardless of whether the main constituent of the L_o_ domain is pSM or DHpSM. To explain these results, we speculated that DHpSM molecules, even at such a low concentration, can form DHpSM-condensed assembly in the SM-rich L_o_ domain due to their higher propensity for intermolecular interaction and thus, they efficiently distribute in the L_o_ domains. If this speculation is valid, the diffusion coefficient of 488neg-DHpSM should be smaller than that of 488neg-pSM in pSM/DOPC/chol because diffusion coefficient is inversely related to the aggregation size^28^. However, in the L_o_ phase, the observed diffusion coefficient of 488neg-DHpSM (0.61 ± 0.07 μm^2^/s, Table 1) was not significantly different from that of 488neg-pSM in pSM/DOPC/chol (0.72 ± 0.06 μm^2^/s, Table 1). This is probably because the concentration of 488neg-DHpSM used for FCS measurements (0.002 mol%) is much lower than that for the determination of the L_o_/L_d_ distribution ratio (0.2 mol%), which may reduce the clustering of 488neg-DHpSM under the FCS measurement conditions.

### Formation of the DHpSM-condensed assembly in the L_o_ domains

To gain evidence for the formation of the DHpSM-condensed assembly inside the pSM-rich L_o_ domains, we prepared quaternary-component GUVs, which contain DHpSM as a minor constituent (1–3.3 mol% of total lipids), and measured diffusion coefficients of 488neg-pSM and 488neg-DHpSM (Table 2). In this experiment, we assumed two scenarios. One is that the minor component DHpSM is miscible in and dispersed into the pSM-rich L_o_ domains at a molecular level and therefore, both 488neg-pSM and 488neg-DHpSM have similar diffusion coefficients. The other is that the minor component DHpSM forms DHpSM-condensed assembly inside the pSM-rich L_o_ domains and thus, 488neg-DHpSM residing in the assembly has a decreased diffusion coefficient compared with 488neg-pSM.

**Table 2:** Diffusion coefficients and radius of lipid assembly in quaternary GUVs.

Molar ratio of pSM/DHpSM/DOPC/chol in GUV	Diffusion coefficient of L_o_ domains (µm^2^/s)^a^		Radius of lipid assembly in L_o_ domains (nm)^b^	
	488neg-pSM	488neg-DHpSM	488neg-pSM	488neg-DHpSM
97: 3: 100: 100	0.71 ± 0.09	0.57 ± 0.06	0.9 ± 0.6	4 ± 2
94: 6: 100: 100	0.74 ± 0.08	0.46 ± 0.07	0.6 ± 0.4	14 ± 9
90: 10: 100: 100	0.65 ± 0.09	0.41 ± 0.06	1.7 ± 1.4	18 ± 9

Diffusion coefficients are presented as mean ± SE. Representative FCS curves are shown in Fig. S3.

^a^The content of fluorescent lipid was 0.002 mol% of total lipids.

^b^The radius of cluster was estimated by diffusion coefficients using Eq. (2).

The results showed that the diffusion coefficient of 488neg-DHpSM was significantly smaller than that of 488neg-pSM (Table 2), supporting the latter scenario. Importantly, 488neg-DHpSM diffusion decreased as the content of DHpSM increased, while 488neg-pSM diffusion was almost constant, irrespective of the content of DHpSM (Table 2). This means that the addition of DHpSM (< 3.3 mol% of all lipids) does not increase the overall order of the pSM-rich L_o_ domains because the diffusion coefficient of 488neg-pSM did not change significantly (Table 2). Therefore, it is most likely that even a small amount of DHpSM (1–3.3 mol% of total lipids) can form the DHpSM-condensed assembly in the pSM-rich L_o_ domains, thereby reducing the diffusion of only DHpSM without enhancing the overall order of the pSM-rich L_o_ domains. In other words, DHpSM molecules are heterogeneously distributed in the pSM-rich L_o_ domains at a molecular level.

We roughly estimated the aggregation sizes of pSM and DHpSM in the L_o_ phase from their diffusion coefficients using the Saffman–Delbrück formula (see “Materials and Methods” section)^29,30^. Consequently, the radius of the DHpSM-condensed assembly (ranging from 4 ± 2 nm to 18 ± 9 nm) is significantly larger than that of pSM (ranging from 0.9 ± 0.6 nm to 1.7 ± 1.4 nm), and the size the DHpSM-condensed assembly is getting larger with the increasing molar ratio of DHpSM (Table 2).

**Figure 4:**
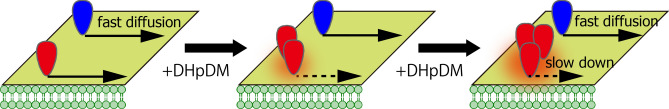
Schematic drawing of the formation of DHpSM-condensed assembly in the L_o_ domain. The blue and red heads correspond to pSM and DHpSM, respectively. Orange membrane regions indicate the DHpSM-condensed assembly (see text for details).

Figure 4 schematically summarizes the results of current diffusion experiments. DHpSM molecules are heterogeneously distributed and form DHpSM-condensed assembly in the pSM-rich L_o_ domains. As the mole ratio of DHpSM increases, the assembly size also increases, which reduces the diffusion of DHpSM. Meanwhile, the diffusion of pSM in the pSM-rich L_o_ domains is hardly affected by the presence of the DHpSM assembly.

**Figure 5:**
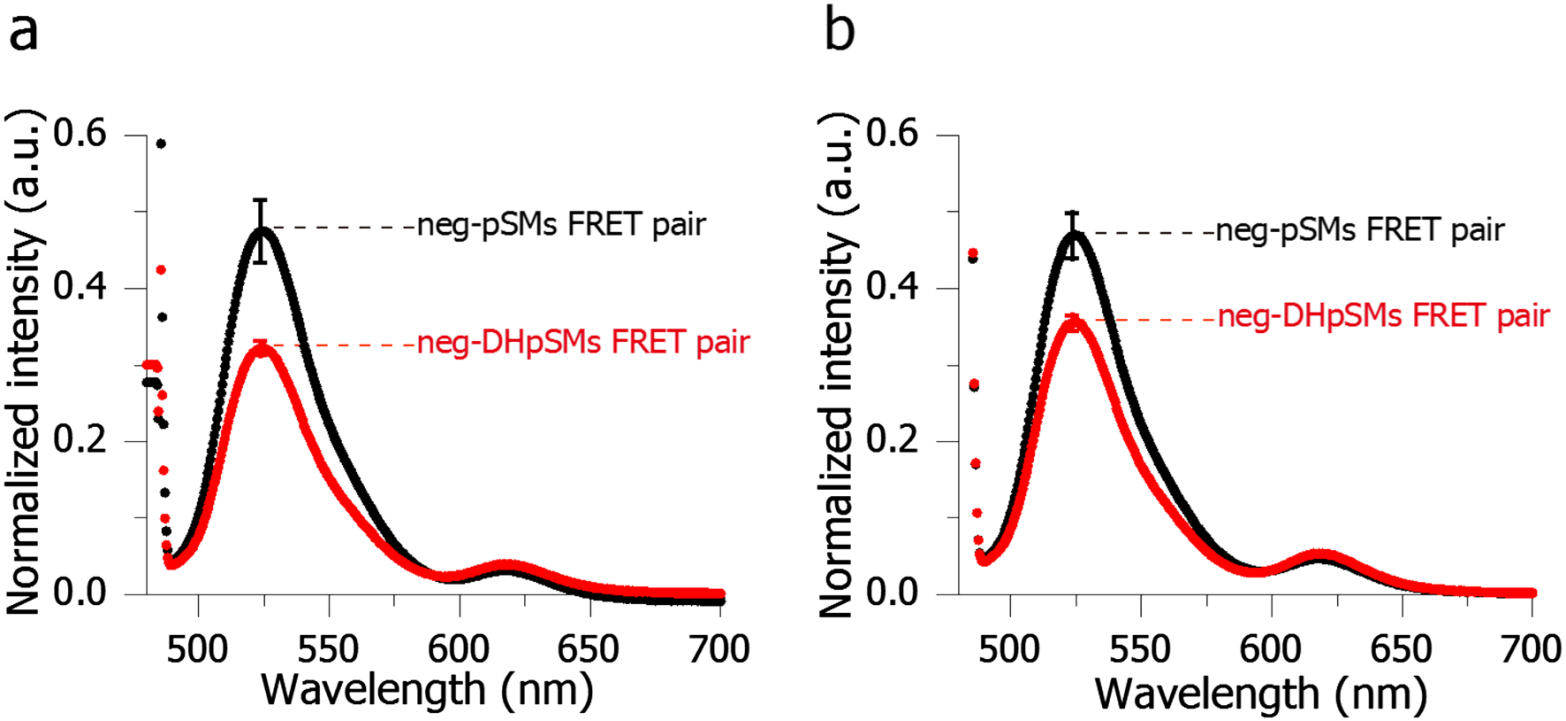
Fluorescent spectra of (**a**) pSM/chol (70:30 by moles) and (**b**) pSM/DHpSM/chol (66.5:3.5:30 by moles) multi-lamellar vesicles. These samples contain 488neg-pSM/594neg-pSM FRET pair (neg-pSMs; black curve) or 488neg-DHpSM/594neg-DHpSM FRET pair (neg-DHpSMs; red curve). The content of each fluorescent SM is 0.4 mol%. Fluorescent measurements were repeated five and four times for the pSM/chol and pSM/DHpSM/chol samples, respectively, and averaged spectra are shown. Error bars show standard errors. The vertical axes indicate the normalized intensity, in which emission peak intensity at 525 nm of the acceptor-free sample was set to 1. Therefore, (1 − normalized peak intensity at 525 nm) corresponds to the FRET efficiency. Panel (**a**) shows that the FRET efficiency for 488neg-pSM/594neg-pSM pair in pSM/chol is ca. 50%. On the other hand, applying Refs.^32,33^ using the Förster-radius (5.7 nm) and acceptor molar ratio (0.4 mol%), the FRET efficiency between randomly distributed donor and acceptor is calculated to be ca. 60%. This is roughly consistent with the observed FRET efficiency for 488neg-pSM/594neg-pSM (ca, 50%), thus supporting their random distribution in pSM-chol membrane.

The formation of the DHpSM-condensed assembly inside the SM-rich L_o_ domains was further confirmed by inter-lipid fluorescent energy transfer (inter-lipid FRET)^11^ using pSM/30 mol% chol binary membrane, which forms the homogeneous L_o_ phase^31^. Because fluorophores, ATTO488 and ATTO594, are an excellent donor–acceptor FRET pair, we included 488neg-pSM/594neg-pSM (neg-pSMs) pair or 488neg-DHpSM/594neg-DHpSM (neg-DHpSMs) pair in the pSM/chol binary membrane and, measured the fluorescence spectra. As shown in Fig. 5a, neg-DHpSMs FRET pair (red curve) showed a weaker emission intensity at 525 nm (namely a stronger FRET quenching) than neg-pSMs FRET pair (black curve). Note that 488neg-pSM and 594neg-pSM should be randomly distributed in this pSM/chol binary membrane because neg-SMs are reported to reproduce the behaviors of native SM in membranes^10^. The random distribution of neg-pSMs in pSM/chol membrane is also supported by Fig. 5a (see Fig. 5 legend for detail). Therefore, a stronger FRET quenching observed for neg-DHpSMs pair indicates the formation of DHpSM-condensed assembly in the pSM/chol L_o_ membrane, thereby supporting the above diffusion data.

In addition, we measured the same spectra using pSM/DHpSM/chol (66.5:3.5:30) membrane (Fig. 5b). In this membrane system, the size of DHpSM assembly should be larger than that in pSM/chol membrane, and thus, the average distance between neg-DHpSMs FRET pair is expected to be elongated as compared in pSM/chol membrane. As expected, a significantly smaller FRET quenching (larger fluorescent intensity) was observed for pSM/DHpSM/chol system (Fig. 5b, red curve) compared with pSM-chol membrane (Fig. 5a, red curve), further confirming the formation of DHpSM cluster in the L_o_ membrane.

### Preferential incorporation of DHpSM into detergent-resistant membrane domains compared to incorporation of pSM in horse erythrocyte ghost membranes

**Figure 6:**
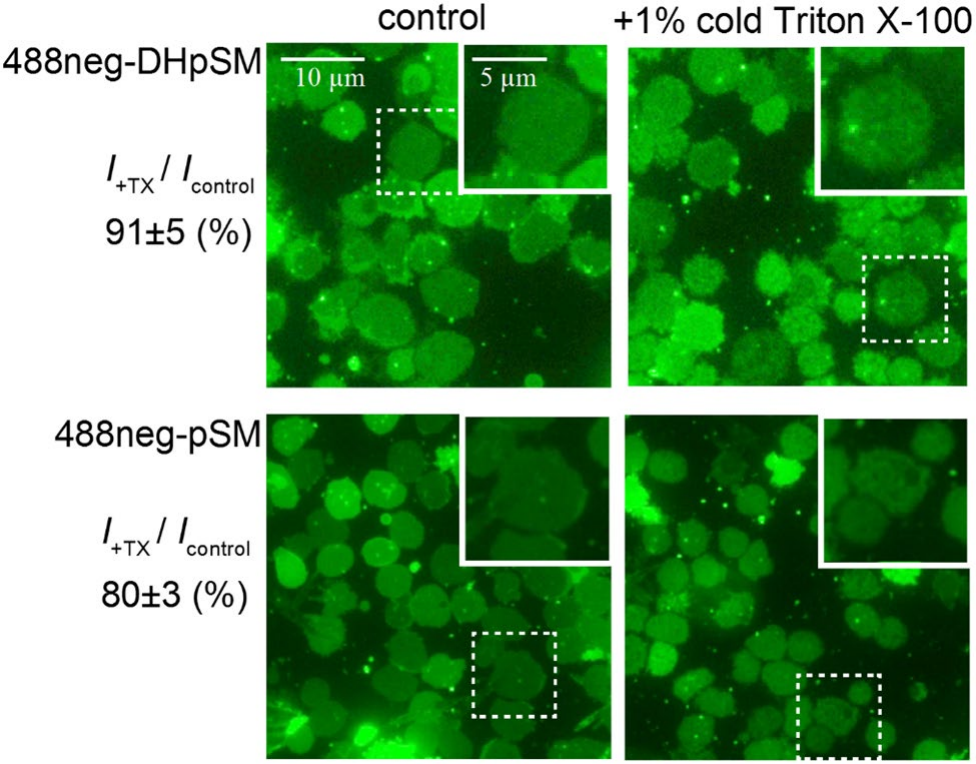
Fluorescent micrographs of (top) 488neg-pSM and (bottom) 488neg-DHpSM-labeled erythrocyte ghosts membranes (left) before and (right) after Triton X-100 treatment. The intensity ratio of after Triton X-100 treatment (*I*_+TX_) to before treatment (*I*_control_) was described at the left-side of the corresponding micrographs. The insertion of each panel shows magnification of the region indicated by the dashed square.

To contribute to the application of fluorescent DHpSM to biological membrane systems, we performed a partitioning assay using horse erythrocyte ghost membranes. In the assay, 488neg-DHpPC or 488neg-pSM was incorporated into the erythrocyte ghost membranes, which were fixed on a glass-bottom dish, and then the membranes were treated with detergent (Triton X-100) at 0 °C. Because the fluid membrane fractions are dissolved in the Triton X-100, only the detergent-resistant membrane (DRM) fractions, which are putative lipid rafts, are obtained on the glass-bottom dish^10^. In fact, erythrocyte ghosts showed rough membrane surfaces after detergent treatment owing to solubilization of the fluid membrane fractions (insertions in Fig. 6). Here, we randomly selected about 50 erythrocyte ghosts before and after detergent treatments, respectively, and compared their average fluorescent intensities (Fig. 6). This analysis shows mole fractions of fluorescent lipid derivatives incorporated into the DRMs. Consequently, although both fluorescent lipids were remained in the DRM fractions, 488neg-DHpPC showed a significantly higher distribution ratio than 488neg-pSM. Although it is reported that DRMs are not completely consistent with lipid rafts^34,35^, this result suggests that DHSM is favorably incorporated into lipid rafts in biomembranes, more than SM; this is consistent with the preceding GUV observation.

## Conclusion

Recently, it has been indicated that DHSM forms raft-like ordered domains more effectively than SM due to its greater potential to induce intermolecular hydrogen bonds^36^. Thus, we expected that DHSM would be a crucial molecule for raft formation and raft-based biological functions. However, owing to the lack of appropriate fluorescent probes, information regarding the partition and dynamic behaviors of DHSM has been difficult to obtain. In the present study, we synthesized promising fluorescent derivatives of DHpSMs based on our recently developed molecular design^10^ and directly observed the distribution and diffusion behavior of DHpSM in L_o_/L_d_ phase-separated GUVs.

Fluorescence microscopy observation disclosed that in L_o_/L_d_ phase-separated GUVs, DHpSM highly favors the L_o_ domains more than SM  (Table 1). The FCS measurements (Table 2) further demonstrated that the diffusion coefficient of 488neg-DHpSM was significantly lower than that of 488neg-pSM in four-component GUVs consisting of SM, DOPC, chol, and a minor amount of DHSM (1–3.3 mol% of total lipids). This strongly suggests that DHpSM, even at a small molar ratio compared with pSM, is not completely miscible in pSM, forming DHpSM-rich aggregation in the pSM-rich L_o_ domain. The formation of the DHpSM-condensed assembly in the L_o_ domain was further confirmed by the inter-lipid FRET measurements, because FRET occurred more efficiently for neg-DHpSMs pair than for neg-pSMs pair in the pSM/chol binary membrane. Since the C4–C5 link in DHSM is a single bond, while it is a double bond in SM, DHSM may be able to adopt a conformation suitable for intermolecular hydrogen-bond formation.

While most previous raft-oriented studies have exclusively used SM as a representative sphingolipid, the disposition of DHSM to the domain formation is not sufficiently understood. This is because DHSM is a minor constituent of sphingolipids and accounts for only 5–10% of all SMs in normal cells^13,14^. However, our current data suggest that such a small amount of DHSM can form the DHSM-condensed assembly in SM-rich lipid rafts, which may have significant roles in membrane-related biological processes. For example, it may not be far-fetched to assume that some membrane proteins are preferentially localized to higher ordered DHSM-rich aggregation in lipid rafts and exert their functions^37^. In this context, this study will lead to a novel understanding of the biological functions and significance of minor-component DHSM in lipid rafts.

## Materials and methods

### Materials

Egg-yolk sphingomyelin and DOPC were purchased from Avanti Polar Lipids (Alabaster AL). Cholesterol was purchased from Sigma Aldrich (St. Louis MO). Palmitoyl-sphingomyelin (pSM) was purified from the egg-yolk SM using high-performance liquid chromatography (HPLC) and the purity was ascertained using thin-layer chromatography, which showed a single spot^38^. DHpSM was prepared from pSM by catalytic hydrogenation. These lipids were dissolved in chloroform/methanol (4:1, v/v) at a concentration of 1 mg/mL and stored at − 30 °C until use. NHS esters of ATTO488 and ATTO594 were purchased from ATTO-TEC GmbH (Siegen, Germany). Texas Red-DPPE (TXred-DPPE) was purchased from Invitrogen (Eugene, OR), and 488neg-DOPC was synthesized following previous report^10^. These dye compounds were dissolved in chloroform/methanol (4:1, v/v) at a concentration of 50 μg/mL or 25 μg/mL and, stored in the dark at − 30 °C until use.

### Preparation of fluorescent lipids

Fluorescent labeled lipids, 488neg- and 594neg-labeled DPPC, pSM, and DHpSM, were prepared from DPPC, pSM, and DHpSM, respectively, as reported previously^10^. In brief, ATTO488- and ATTO594-neg-azides were conjugated to propargyl-DPPC, -pSM, and -DHpSM, all of which were prepared following the previously reported method^39^, using the Huisgen reaction, providing the objective compounds.

- *488neg-pSM* red solid; R_f_ = 0.42 (CHCl_3_/MeOH/H_2_O = 6/4/1); MS(ESI-TOF) m/z [M + 3Na − H]^2+^ calc. for C88H145N9Na3O25PS2^2+^, 945.9604; found 945.9661.
- *594neg-pSM* blue solid; R_f_ = 0.76 (CHCl_3_/MeOH/H_2_O = 13/9/1); MS(ESI-TOF) m/z [M + 3Na − H]^2+^ calc. for C104H169N9Na3O25PS2^2+^, 1,054.0545; found 1,054.0573.
- *488neg-DHpSM* red solid; R_f_ = 0.49 (CHCl_3_/MeOH/H_2_O = 6:4:1); MS(ESI-TOF) m/z [M + 3Na − H]^2+^ calc. for C88H147N9Na3O25PS2^2+^, 946.9685; found 946.9686.
- *594neg-DHpSM* blue solid; R_f_ = 0.68 (CHCl_3_/MeOH/H_2_O = 8/1.9/0.1); MS(ESI-TOF) m/z [M + 3Na − H]^2+^ calc. for C104H171N9Na3O25PS2^2+^, 1,055.0624; found 1,055.0694.
- *488neg-DPPC* red solid; R_f_ = 0.48 (CHCl_3_/MeOH/H_2_O = 6/4/1); MS(ESI-TOF) m/z [M + 3Na − H]^2+^ calc. for C89H146N8Na3O27PS2^2+^, 961.4579; found 961.4556.
- *594neg-DPPC* blue solid; R_f_ = 0.48 (CHCl_3_/MeOH/H_2_O = 6/4/1); MS(ESI-TOF) m/z [M + 3Na − H]^2+^ calc. for C105H170N8Na3O27PS2^2+^, 1,069.5518; found 1,069.5599.

### Giant unilamellar vesicle (GUV) preparation and fluorescent observation

GUVs were prepared using an electroformation method^40^. Briefly, appropriate amounts of the lipid solutions were mixed in a glass vial (1 mg/mL), to which small amounts of fluorescent lipid analogs (0.2 mol% of total lipids) were added. The 10-μL aliquot of the lipid solution was spread on the surface of electrodes, platinum wires (100 μm diameter), and dried under vacuum for more than 12 h. Then parallel aligned electrodes were put into Milli-Q water sandwiched between two cover glasses (24 mm × 60 mm, 0.12–0.17 mm thickness) using an open-square-shaped rubber spacer. This chamber was fixed on a temperature-controlled aluminum block kept at 70 °C (Sahara 310, Rocker Scientific Co., Ltd., Taipei, Taiwan). The sample was incubated for 60 min, applying a low frequency alternating current (AC) field (sinusoidal wave function, 10 Vpp, 10 Hz), by a function generator (20 MHz function/arbitrary waveform function generator, Agilent, Santa Clara CA). The GUVs were then cooled to 25 °C. Fluorescent observation was carried out using the BZ-X700 (Keyence, Osaka, Japan) with an air objective lens (Plan Apoλ, 60 ×, N.A. 0.95, Nikon, Tokyo, Japan). The excitation (470 nm) and detection (525 nm) wavelengths were selected by dichroic mirrors, OP-87763 (Keyence, Osaka, Japan).

### Inter-lipid fluorescent resonance energy transfer (FRET) measurements

Multi-lamellar vesicles (MLVs) of pSM/chol (70:30 by moles) and pSM/DHpSM/chol (66.5:3.5:30 by moles) containing 0.4 mol% FRET donor, 488neg-pSM or 488neg-DHpSM, and 0.4 mol% FRET acceptor, 594neg-pSM or 594neg-DHpSM, were prepared by a conventional method^41^. Briefly, appropriate amounts of SM, DHpSM and chol (total 0.100 μmol) were dissolved in chloroform/methanol (4:1 v/v) in the presence of the FRET donor and acceptor and dried under N_2_ flow. The remaining organic solvent was completely removed in vacuo for > 12 h. The dried lipid films were suspended in 1 mL of Milli-Q water at 80 °C with intermittent vortexing to form MLVs. The MLV suspensions were equilibrated for 30 min at room temperature for the subsequent FRET measurements. For preparation of the acceptor-free samples, the same procedure was adopted except getting rid of the FRET acceptor. Excitation was applied at 480 nm and the emission spectra were obtained from 450 to 700 nm at an interval step of 5 nm using a FP-8300 spectrofluorometer (JASCO Corp., Tokyo, Japan). The temperature was maintained at 25 °C with a CTU-100 water circulator (JASCO Corp., Tokyo, Japan).

### Fluorescent correlation spectroscopy (FCS)

FCS measurements at GUV surfaces were performed at 23 °C with a confocal microscope (LSM880, Leica microsystems, Wetzlar, Germany), with a water immersion objective lens (C-Apochomat 40 × /1.2 W Korr M27), following the protocol published previously^42,43^. The diffusion coefficient, *D*, was obtained by fitting the autocorrelation function of the time-dependent changes of the signal intensities of fluorescent probe molecules in diffraction-limited spots, *G*(*τ*), with the following equation for two-dimensional simple Brownian diffusion:

1 $${\text{G}}\left( \tau \right) = \frac{1}{N}\left\{ {\frac{1}{{1 + \left( {\frac{4D\tau }{{W_{0}^{2} }}} \right)}}} \right\},$$

where *N* is the average number of fluorescent particles in the detection area. The beam waist (radius) in the focal plane *w*_0_ (= 0.185 μm) was calibrated with Rhodamine 6G, for which the diffusion coefficient is widely known. *D* is the diffusion coefficient, and *τ* is the delay time. When the FCS measurements were performed at the L_o_ and L_d_ domains, 0.002 mol% and 0.004 mol% fluorescent lipids were added to the lipid mixture, respectively. The obtained FCS curves were analyzed by the software package ZEN 2.

### Size estimation of the DHpSM and pSM clusters

Cluster sizes of pSM and DHpSM in the L_o_ phase were estimated by diffusion coefficients of 488neg-pSM and 488neg-DHpSM, respectively. Relationship between the radius *R* of cylindrical membrane inclusions, such as lipids and proteins, and their diffusion coefficients *D* was expressed by the Saffman–Delbrück formula^29,30^.

2 $$D = \frac{kT}{{4\pi \eta h}}\left( {ln\frac{\eta h}{{\eta_{w} R}} - \gamma } \right).$$

Here *k*, *T* and *γ* are Boltzmann’s constant (1.38 × 10^−23^ J/K), temperature (295 K) and Euler’s constant (0.5772), respectively. *η* (= 0.81 Pa s) and *η*_w_ (= 1 × 10^−3^ Pa s) are viscosities of the L_o_ membrane formed in brain-SM/DOPC/chol^30^ and water^30^, respectively. *h* (~ 4.5 nm) is membrane thickness of the L_o_ phase consisting of pSM/50 mol% chol^44^. In this calculation, we assumed that small amounts of DHpSM (< 3.3 mol% of total lipids) did not affect the viscosity and thickness of the L_o_ membrane.

### Preparation of horse erythrocyte ghost membranes and incorporation of fluorescent lipid analogs

Horse erythrocyte ghost membranes were prepared essentially as previously reported^45^. Briefly, erythrocytes obtained from horses were pelleted by centrifugation, and after the removal of the buffy coat, 100 μL of the erythrocyte pellet was resuspended in 900 μL prechilled hypotonic 5 mM NaH_2_PO_4_-Na_2_HPO_4_ buffer (pH 8.0; 5P8) and incubated on ice for 20 min, to induce cell lysis. The cells were then washed four times with a 10 × volume of prechilled 5P8 buffer, by resuspension/centrifugation (12,000 × *g*, 10 min, 4 °C). Then, 5P8 buffer was replaced by phosphate-buffered saline (PBS; pH 7.6) buffer to form erythrocyte ghosts.

The erythrocyte ghost membranes were labeled with 488neg-pSM and 488neg-DHpSM as previously reported^10^. Briefly, erythrocyte ghosts were incubated with 1 μM fluorescent lipid analog in PBS buffer at 37 °C for 20 min in the dark. In order to remove the fluorescent lipid analog which was not incorporated into membranes, the samples were washed four times with a 10 × volume of PBS by resuspension/centrifugation (12,000 × *g*, 10 min, 4 °C). Finally, the sample was diluted in PBS at ~ 10% (v/v). For the subsequent microscopic observation, the erythrocyte ghosts were fixed on poly-*L*-lysine-coated glass-base dishes. For Triton extraction, the ghost membranes fixed on the glass surface were incubated with 1% cold Triton X-100 at 0 °C for 20 min and then, washed three times with prechilled PBS.

## Supplementary information

Supplementary Information.
