# Supplementary material for: Assembly formation of minor dihydrosphingomyelin in sphingomyelin-rich ordered membrane domains
Source: Sci Rep. 2020 Jul 16;10:11794. doi: 10.1038/s41598-020-68688-7 (PMC7366691; doi:10.1038/s41598-020-68688-7)
Supplement: Supplementary file 1 — Supplementary Information. [file 41598_2020_68688_MOESM1_ESM.pdf]

## Supplementary Information

# Assembly formation of minor dihydrosphingomyelin in sphingomyelin-rich ordered membrane domains

Masanao Kinoshita*, Takumi Kyo, Nobuaki Matsumori*

Department of Chemistry, Graduate School of Science, Kyushu University

Motooka 744, Nishi-ku, Fukuoka 819-0395, Japan

*Corresponding authors

Masanao Kinoshita, Nobuaki Matsumori

Tel/fax: +81-92-802-4148 (MK), +81-92-802-4153 (NM)

e-mail: kinoshi@chem.kyushu-univ.jp (MK), matsmori@chem.kyushu-univ.jp (NM)

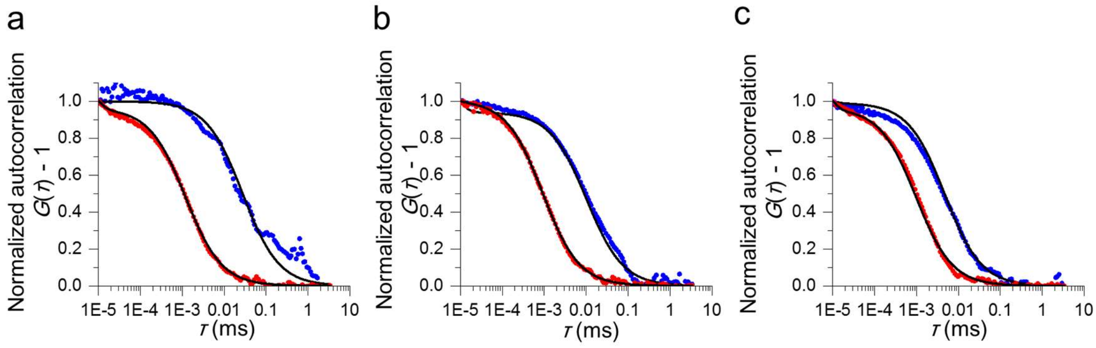


**Figure S1**. Representative FCS curves of (a) 488neg-DHpSM, (b) 488neg-pSM, and (c) 488neg-DPPC in GUVs consisting of DHpSM/DOPC/chol (1:1:1 molar ratio), pSM/DOPC/chol (1:1:1 molar ratio), and DPPC/DOPC/chol (1:1:1 molar ratio), respectively. Red and blue curves show normalized autocorrelations in the $L_d$ and $L_o$ phases, respectively. Black curves show fitting results.

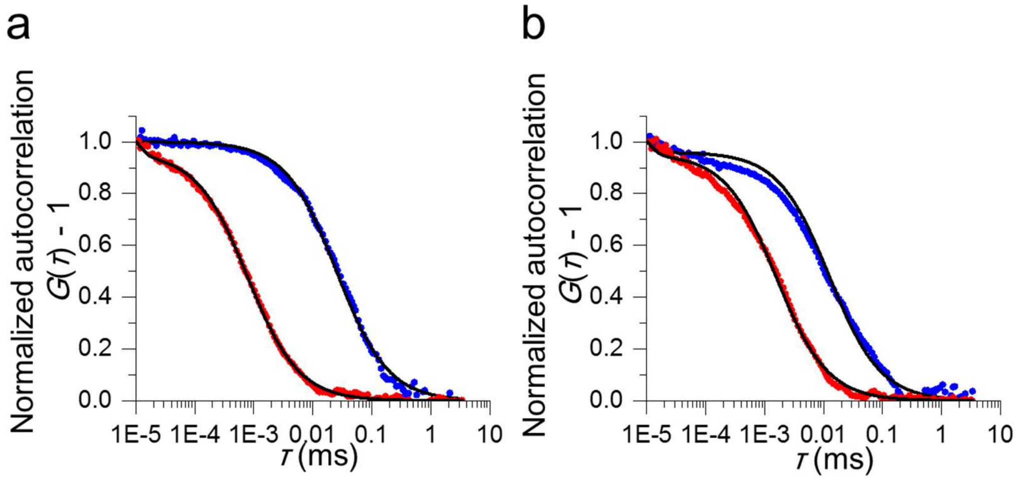


**Figure S2**. Representative FCS curves of (a) 488neg-pSM and (b) 488neg-pSM in GUVs consisting of DHpSM/DOPC/chol (1:1:1 molar ratio) and pSM/DOPC/chol (1:1:1 molar ratio), respectively. Red and blue curves show normalized autocorrelations in the $L_d$ and $L_o$ phases, respectively. Black curves show fitting results.

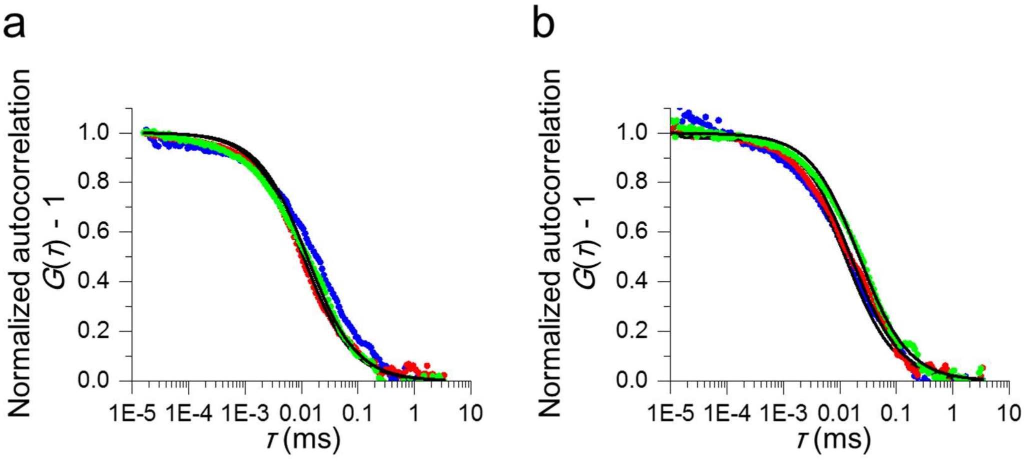


**Figure S3**. Representative FCS curves of (a) 488neg-pSM and (b) 488neg-DHpSM in GUVs consisting of pSM/DHpSM/DOPC/chol in the molar ratio of (blue) 97:3:100:100, (red) 94:6:100:100, and (green) 90:10:100:100, respectively. These data were obtained in the $L_o$ phase. Black curves show fitting results.
